# Velocity of Lordosis Angle during Spinal Flexion and Extension

**DOI:** 10.1371/journal.pone.0050135

**Published:** 2012-11-16

**Authors:** Tobias Consmüller, Antonius Rohlmann, Daniel Weinland, Claudia Druschel, Georg N. Duda, William R. Taylor

**Affiliations:** 1 Epionics Medical GmbH, Potsdam, Germany; 2 Julius Wolff Institute, Charité – Universitätsmedizin Berlin, Berlin, Germany; 3 Center for Musculoskeletal Surgery, Charité – Universitätsmedizin Berlin, Berlin, Germany; University of South Australia, Australia

## Abstract

The importance of functional parameters for evaluating the severity of low back pain is gaining clinical recognition, with evidence suggesting that the angular velocity of lordosis is critical for identification of musculoskeletal deficits. However, there is a lack of data regarding the range of functional kinematics (RoKs), particularly which include the changing shape and curvature of the spine. We address this deficit by characterising the angular velocity of lordosis throughout the thoracolumbar spine according to age and gender. The velocity of lumbar back shape changes was measured using Epionics SPINE during maximum flexion and extension activities in 429 asymptomatic volunteers. The difference between maximum positive and negative velocities represented the RoKs. The mean RoKs for flexion decreased with age; 114°/s (20–35 years), 100°/s (36–50 years) and 83°/s (51–75 years). For extension, the corresponding mean RoKs were 73°/s, 57°/s and 47°/s. ANCOVA analyses revealed that age and gender had the largest influence on the RoKs (p<0.05). The Epionics SPINE system allows the rapid assessment of functional kinematics in the lumbar spine. The results of this study now serve as normative data for comparison to patients with spinal pathology or after surgical treatment.

## Introduction

Low back pain is one of the most common diseases in western industrialised countries [1]; [2]. Besides the relief of pain, therapeutical measures focus on the conservation and improvement of the subject's functional capacity. Recently, clinical attention has been drawn to assessing the kinematics of changes in spinal shape, which have been shown to provide a greater distinction between patients with low back pain pathology and asymptomatic subjects than measures of e.g. range of motion alone. In this respect, Marras and co-workers demonstrated the importance of dynamics during functional activities by investigating 16 low back pain patients and 18 asymptomatic volunteers using the Ady-Hall lumbar monitor [3]. While they found a reduction of 10% in the range of motion during flexion in low back pain patients compared to healthy volunteers, the significant reduction of 50% in angular velocity indicated a much clearer biomarker for low back pain. More importantly, during extension, the angular velocity of patients was reduced by more than 90%. Further evidence demonstrating the importance of dynamic measures was provided by McGregor and co-workers [4], who examined 20 low back pain patients and 20 healthy volunteers using the CA-6000 [5], similarly concluding that the velocity of spinal flexion in the sagittal plane was a clear target for functional identification of pathology.

A number of measurement tools exist for the objective estimation of the lumbar spinés range of motion (RoM), with some offering the change of back shape with respect to time, including Vicon [6], ZooMS [7], Formetric 4D [8], 3space [9], 3D-SpineMoveGuard [10], fibre-optic sensor [11] and inertial measurement units [12]. However, numeric data for dynamic measures of spinal kinematics are only available for the Ady-Hall lumbar monitor, the CA-6000 and the lumbar motion monitor [3]; [4]; [13]–[15]. Widespread accessibility to rapid and mobile approaches for assessing spinal kinematics is, however, critical for these important measures to be considered for aiding clinical decision making.

The so-called range of functional kinematics (RoKs) provides a measure of the maximum and minimum flexion and extension velocities. Normative data has been published for the measurement tools CA-6000 and lumbar motion monitor [16]; [17]. While the potentiometer link arm of the CA-6000 is positioned at the thoracolumbar joint and at the level of the spina iliaca superior posterior, the lumbar motion monitor uses an electro-goniometer that is attached to the shoulder and pelvis. Thus, these devices measure the velocity for different regions of the back, but are unable to consider the dynamic shape of the back, including the changing curvature at different regions of the spine. In order to allow the formation of normative reference data for clinical usage, where pain and musculoskeletal deficits occur at different heights, complete datasets of dynamic back shape are required, but remain to be established.

The measurement tool Epionics SPINE is an advancement of the former SpineDMS system [18], and allows the dynamic assessment of the shape of the thoracolumbar spine in a rapid and subject specific manner based on strain gauge technology. While age, gender and body-height dependent normative data for back shape and RoM have been determined for this device, no repository of normative data exists for the maximum velocities of lumbar spine movements in the sagittal plane, i.e. flexion and extension.

With the goal of establishing normative data for comparison against patients with spinal pathology or after surgical treatment, the aim of this study was to determine the velocities during changes of lordosis angle for movements in the sagittal plane in healthy volunteers, and therefore quantify changes in dynamic back shape. Furthermore, we aimed to characterise back shape such that parameters of the lumbar functional capacity with respect to individual factors such as age and gender can be derived.

## Materials and Methods

### Ethics Statement

The study was approved by the Ethics Committee of the Charité – Universitätsmedizin Berlin (registry number EA4/011/10), and each volunteer provided written informed consent to participate.

### Subjects

The lumbar spine movements of 429 asymptomatic volunteers (231 females, mean age 40.0±15.2, 198 males, mean age 39.3±13.6 years) were assessed. Inclusion criteria for the study were an age between 20 and 75 years, the absence of back pain in the previous 6 months, and no previous spinal surgery. For the analysis of age dependency, volunteers were divided into classes of 20–35, 36–50 and 51–75 years (189, 146 and 94 persons respectively).

### Measuring system

**Figure 1:**
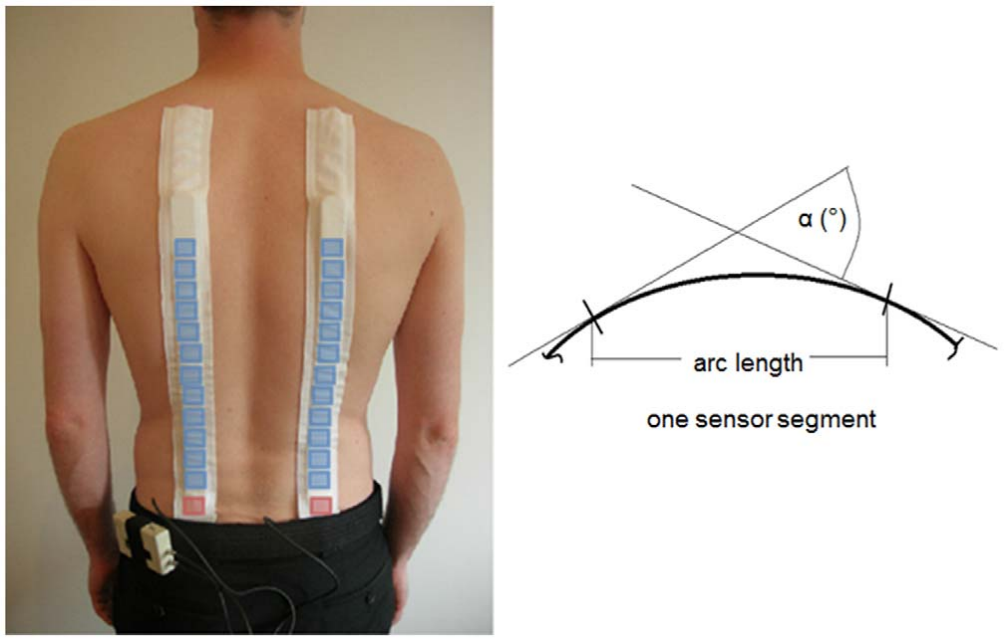
Measurement system. Epionics SPINE system with schematic positions of bending sensor segments (blue) and acceleration sensors (red), (left). A schematic display of the definition of angle α is shown for a single exemplary bending sensor segment (right).

Measurements were conducted using Epionics SPINE (Epionics Medical GmbH, Potsdam, Germany), which allows the temporal assessment of back shape in the region of the thoracolumbar spine for motions in the sagittal plane [19]. The system has been described in detail elsewhere [19], but a brief summary is provided here: Two flexible sensor strips are fixed paravertebrally to the spine using special hollow plasters (Figure 1, left). The strips are placed a distance of 5 cm from the mid-sagittal plane and the lowest sensor segment is positioned relative to the spina iliaca posterior superior. Each sensor strip assesses the curvature of the back shape along the 12 connected segments by measuring bending of the segments relative to one another using a series of strain gauge measurements (Figure 1, right). The sensors are connected via cables to a memory unit, which provides storage of the data, which is collected at 50 Hz, as well as a power supply. The validity and reliability of the measurement tool, as well as normative data for spinal RoMs, have been published elsewhere [18]; [19].

### Measurement protocol

The volunteers performed standard upper body movement choreographies after watching a video, which explained and demonstrated the requested movements. Each subject was asked to perform maximum spinal flexion and extension exercises with extended knees each five times. Between the exercises, each subject's upright standing posture was assessed as a reference position. No instructions were provided to the subjects concerning the velocity of the upper body movements. Thus, all volunteers performed the movements at their preferred speed, which is known to produce more consistent results of motion characteristics (RoM as well as RoKs) than pre-defined slow or maximum speeds [20].

### Data analysis

In this study, measurement results were averaged over the left and right sensor strip since only movements in the sagittal plane were considered and the sensor strips were attached symmetrically to the spine [19]. The area of the sensor strips that covered the lumbar lordosis was identified individually for each volunteer as the range of segments that have negative bending during upright standing. The angles of these segments were then summed at every time frame, and the derivative with respect to time, calculated using the Savitzky-golay differentiation filter in the Matlab suite (The Mathworks Inc., Natick, MA, USA), was used to compute the angular velocities. The velocities presented are the mean peak velocities reached during the five repetitions of descending or ascending movement.

### Description of the functional capacity

The functional capacity was considered to consist of a combination of the maximum RoM and the maximum RoKs. At each measurement time point, the lordosis angle was therefore computed and the corresponding velocities were calculated using the derivative with respect to time. Velocities were considered positive during movement in anterior direction (descending during flexion and ascending during extension) and negative during movement in the posterior direction (ascending during flexion and descending during extension).

In order to understand the variation of dynamic metrics, an average curve of the volunteers in different age classes was constructed by applying a dynamic time warping procedure to the curves of each volunteer [21]. This temporal standardisation allowed a comparison of the repeated movements for different volunteers at individual instances of time.

The lordosis variation for the RoM was computed as the difference of the lordosis angle at maximum flexion and maximum extension respectively, and for the RoKs as the difference between the maximum (+ve) and minimum (−ve) velocities during flexion and extension.

### Statistics

For the determination of number of volunteers required to determine spinal RoKs representative of the population, a power analysis was conducted with nQuery 7.0 (Statistical Solutions Inc., Saugus, MA, USA), using 2-sided 95% confidence interval. Mean RoKs of 106°/s and a standard deviation of 33°/s, obtained from the results of the pilot study, indicated that at least 336 volunteers are required to create a normative database for lumbar RoKs. A covariance analysis (ANCOVA) was used to identify which of the individual parameters (age, gender, BMI (body mass index), body height and weight) played a dominant role on the maximum velocities reached during flexion and extension. Analyses of variance (two-way ANOVA) were used to examine interactions and the statistical variation between groups. The significance level was set to 0.05. The arithmetic means and standard deviations were computed from the maximum velocities for each of the different volunteer groups. Statistical analyses were performed using Matlab (The Mathworks Inc., Natick, MA, USA) and SPSS 19 (IBM, Armonk, NY, USA).

## Results

### Flexion and Extension

**Figure 2:**
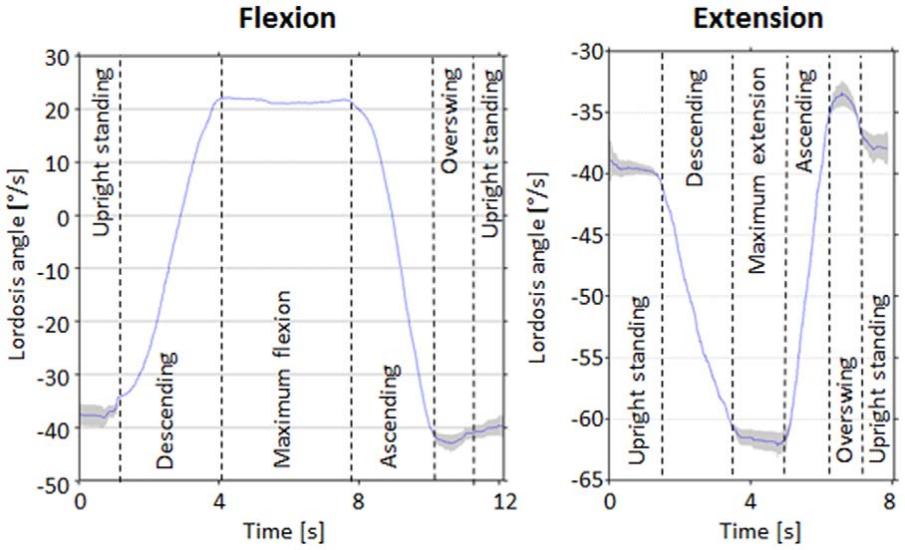
Lordosis angle versus time. Exemplary mean curvature of lordosis angle versus time for one volunteer during a flexion (left) and extension exercise (right). The grey area represents one standard deviation of repeated movements.

All 429 subjects were able to complete the full movement analysis program without incident. During the flexion movement in general, the magnitude of the negative lordosis angle reduced initially and reached a positive angle before returning to approximately its initial value in the static standing position (Figure 2, left). During the extension activity, the magnitude of the lordosis angle first increased to a maximum angle before returning to the baseline (Figure 2, right). Variations in the repetitions are visible during upright standing, particularly during the over-swing and maximum deflection phases.

**Figure 3:**
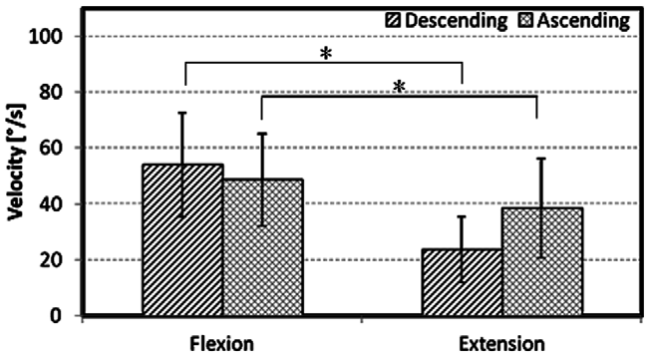
Maximum lordosis angle velocities during flexion and extension. Means and standard deviations of the maximum reached lordosis angle velocities at descending and ascending movement during maximum flexion and extension for all volunteers. Significant differences (* p<0.05) appear between flexion descending-ascending, extension descending-ascending, flexion descending-extension descending, and flexion ascending-extension ascending.

The slope of these flexion-extension curves were determined to provide the corresponding angular velocity, where a larger slope was associated with a higher velocity. During the flexion and extension exercises, two extreme values arose: the first during movement into maximum deflection (descending) and the second while returning to the initial position (ascending). For flexion, the average velocity during the descending movement was significantly higher (p<0.05) than during the ascending movement (Figure 3). During the extension activity, the relative velocity of extension and flexion were reversed such that the velocity during the ascending movement was higher (p<0.05). Significant differences were also found when comparing the velocities of descending flexion and extension and ascending flexion and extension.

### Age and gender differences during flexion

**Figure 4:**
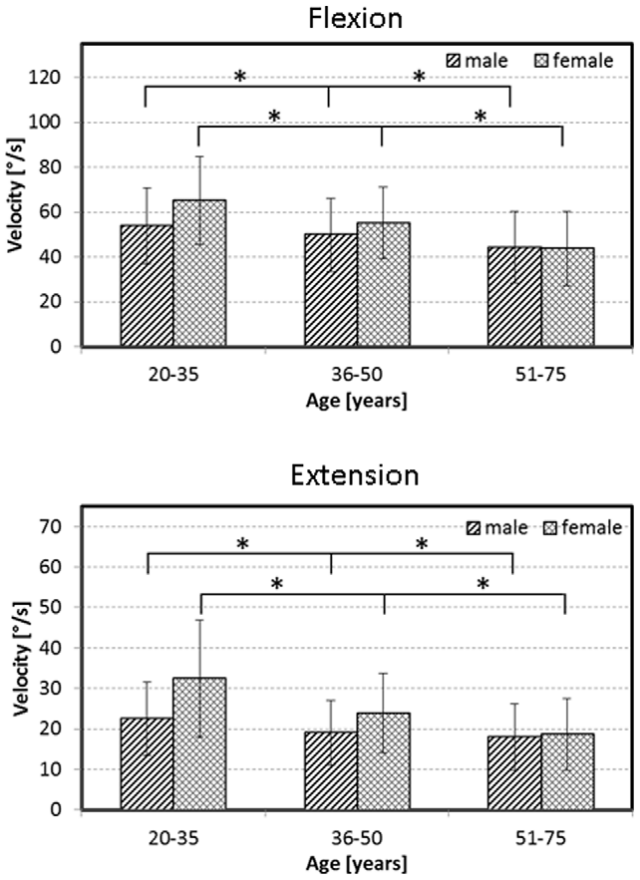
Influence of age and gender on maximum lordosis angle velocity. Means and standard deviations of the maximum lordosis angle velocities during the descending movement into maximum flexion (top) and extension (bottom), displayed according to age and gender. An asterisk (*) indicates statistical significance at the 5% level.

The maximum velocities for both the descending and ascending movements during flexion decreased significantly with increasing age (Figure 4, top, p<0.05). In the youngest age group, females showed higher velocities during flexion (both descending and ascending) compared to the corresponding velocities in males (p<0.05). However, the angular velocities during flexion for females aged between 36 and 50 were higher than in the corresponding males for ascending movements only (p<0.05). No significant differences were found in the oldest age groups.

### Age and gender differences during extension

During extension, a progressive decrease of the angular velocities during both descending and ascending movements was also observed with increasing age (Figure 4, bottom, p<0.05). Males aged between 20 and 35, as well as 36 and 50 years were significantly slower in both descending and ascending (p<0.05). Again, for the highest age group no significant differences were apparent.

### Description of functional capacity

**Figure 5:**
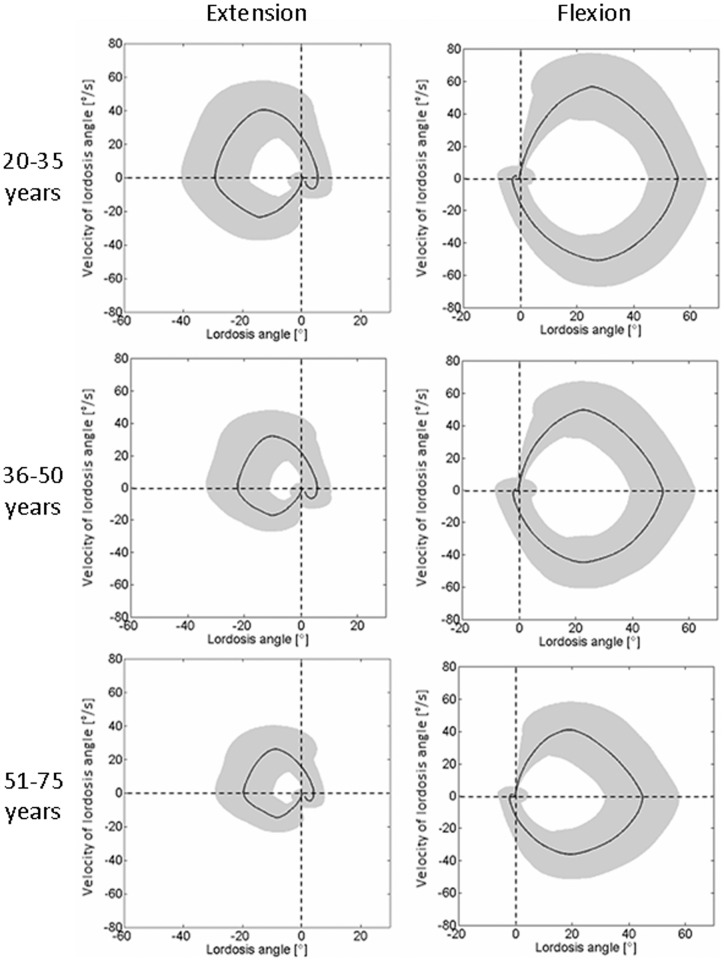
Lordosis angle versus velocity of lordosis angle. Averaged lordosis angle, normalized to the upright standing lordosis, versus the velocity of lordosis angle for extension (left) and flexion (right) depending on age. All figures progress in a clockwise direction. The grey area represents one standard deviation.

An analysis of the lordosis angle compared to the angular velocity resulted in circular patterns for the flexion and extension exercises (Figure 5). By normalizing the lordosis angle to the upright standing position, the curves began at 0° and 0°/s. For the maximum flexion/extension angle, the velocity was also zero. The maximum magnitude of the velocity occurred mostly in the middle region of each movement. With increasing age, the maximum angles as well as the maximum velocities became smaller for both flexion and extension exercises. When returning to the initial position after each activity, an over-swing was normally observed, in which the motion was slightly more than required to return to their baseline position. The RoM and RoKs for the different age groups is provided in Table 1.

**Table 1:** Means of range of motion (RoM) and range of functional kinematics (RoKs) for flexion and extension dependent upon age grouping.

Age	RoM Extension [°]	RoM Flexion [°]	RoKs Extension [°/s]	RoKs Flexion [°/s]
20–35 years	29.7±11.0	54.0±9.3	73.2±31.1	114.1±34.6
36–50 years	22.4±11.2	50.3±10.1	57.4±25.3	100.3±31.8
51–75 years	19.5±9.3	45.1±12.6	47.1±23.1	82.7±30.9

### ANCOVA and ANOVA analyses

The ANCOVA analysis revealed that age and gender had the main influence on the maximum angular velocities reached during flexion and extension exercises for both descending as well as for ascending (Table 2). The ANOVA analysis to determine the interactions between the variables with a major influence on the maximum velocities (age and gender) showed a significant interaction between all velocities except the minimum velocity during flexion (Table 3).

**Table 2:** Results of the ANCOVA analysis showing the importance of age, gender, body mass index (BMI), height, and weight in determining the maximum velocities during flexion and extension exercises, bold values indicate statistical significance at p<0.05.

	F	*p*	Eta-squared
Maximum velocity during flexion (descent)			
Age	**52.32**	**<0.01**	**0.110**
Gender	4.05	0.05	0.009
Height	0.16	0.69	0.000
BMI	0.14	0.70	0.000
Weight	0.03	0.86	0.000
Minimum velocity during flexion (ascent)			
Age	**51.66**	**<0.01**	**0.109**
Gender	**8.03**	**<0.01**	**0.019**
Height	0.21	0.64	0.001
Weight	0.08	0.78	0.000
BMI	0.01	0.94	0.000
Maximum velocity during extension (descent)			
Age	**71.55**	**<0.01**	**0.145**
Gender	**9.34**	**<0.01**	**0.022**
BMI	0.27	0.61	0.001
Weight	0.17	0.68	0.000
Height	0.09	0.77	0.000
Minimum velocity during extension (ascent)			
Age	**60.63**	**<0.01**	**0.125**
Gender	**18.44**	**<0.01**	**0.042**
Height	0.79	0.38	0.002
Weight	0.66	0.42	0.002
BMI	0.60	0.44	0.001

The ANCOVA degree of freedom was 1 in all cases.

**Table 3:** Results of the two-way ANOVA analysis showing the interaction effects of different velocity measures.

	F	*p*	Eta-squared
Maximum velocity during flexion (descent)	3.77	0.02^*^	0.018
Minimum velocity during flexion (ascent)	2.83	0.60	0.013
Maximum velocity during extension (descent)	5.10	<0.01^*^	0.024
Minimum velocity during extension (ascent)	6.41	<0.01^*^	0.029

A major influence was observed for the maximum velocities of flexion and extension. The degree of freedom was 2 in all cases. ^*^ indicates statistical significance at p<0.05.

## Discussion

Low back pain is often associated with dynamic activities of patients, however the characteristics of dynamic movements, specifically their velocities and changes of velocities are not well known. Differences in dynamic metrics during spinal motion, particularly the angular velocity during flexion and extension movement, are known to play a critical role for differentiating asymptomatic subjects from those with pathological low back pain [3]; [4]. The use of novel technologies for the assessment of dynamic back shape [5]; [17] now allows quantification of the key kinematic characteristics between these groups and can aid towards understanding the role of pathology on functional outcome. This study has presented normative data measured in a collective of 429 asymptomatic volunteers, and provides clear evidence that age and gender have a dominant influence on the maximum angular velocity of the lumbar spine, as well as the range of functional dynamics.

The parameters age and gender had the main influence on the variation of maximum angular velocity during flexion and extension exercises. These parameters were also identified to have the main influence on the variation of range of motion in the sagittal plane [19], which agrees well with earlier findings [17]. Furthermore, interactions between age and gender at different RoMs have been documented previously [22]. The youngest and mid-aged females showed surprisingly higher angular velocities during flexion and extension than their male counterparts, even though there were no gender specific significant differences in age groupings (20–35 years: p = 0.523, 36–50 years: p = 0.647, 51–75 years: p = 0.041). Furthermore, the RoKs of males and females converged with increasing age. Here, while a comparison with pathological movement patterns was not possible within the confines of this study, these normative data do provide a basis for understanding pathology and the expected limitations in patient cohorts.

The resulting velocities for movements in the sagittal plane are very similar to the results of Marras and co-workers [3]. Their volunteers, which were comparable to the youngest and middle aged groups of the current collective, also moved faster in the anterior than in the posterior direction. Moreover, volunteers descended into maximum extension slower than they ascended to upright standing from full flexion. One possible explanation for this relative difference in velocity is that the volunteers maintained slower movement patterns during their approach towards maximum extension in order to reduced their out of balance forces and therefore their risk of falling [23]. Any subsequent movements in the forwards direction to return to upright standing could then happen faster, possibly due to the lever arm offered by the feet for maintaining balance. Although these findings are partly contrary to the findings of McGregor and co-workers [4]; [17] whose volunteers for the most part reached higher velocities during backward motion towards maximum extension than during forward motion to upright standing, no age dependent normative data has been published until now for movements without resistance. From the results of the current study, it seems that this important factor in modifying the speed of spinal movement patterns might be the key to understanding differences between study cohorts [16].

The quantification of the lordosis angle and velocity of lordosis angle offers a multidimensional evaluation of the spinal functional capacity. The computation of differences between minimum and maximum allows the evaluation of a subject's function on the basis of just a few parameters. Although no patients have been examined in this study, the characterisation of functional and kinematic data presented here and previously [5]; [17] will now allow a reference for assessing patients (Figure 4), where it is expected that deficits in RoM and RoKs will be detectable [3]; [4]. Whether the analysis of functional and kinematic data alone will be sufficient to determine e.g. location or extent of a musculoskeletal deficit of the spine, remains to be investigated, but current indications are that such non-invasive data could indeed aid clinical diagnosis and decision making processes.

In this study, no information about the targeted velocity of motion was provided to the volunteers prior to the measurements. As a result, some asymptomatic volunteers conducted the exercises slowly and with caution. Here, this subject specific response might have proved beneficial to the reliability of the study, since each volunteer's preferred pace is known to be the best choice for consistent results [20]. Furthermore, the Epionics SPINE measurement tool is attached to the back in the thoracolumbar region. As a consequence, it could be expected that subjects with a high BMI will produce large variations, but the results of this study indicate that BMI is consistently a non-dominant factor in determining differences between RoKs (Table 2). On the other hand, repetitions of exercises seemed to be highly reproducible between measurements (Figure 2), with greater levels of variation observed when reaching maximum extension – a result that is presumably associated with greater instability in this position. In this respect, additional studies into the reproducibility of movement patterns, particularly the extremes of motion, will be addressed in future studies.

The assessment of RoM and RoKs of the upper body, and therefore an evaluation of physical function, has been enabled using Epionics SPINE in an easy and non-invasive manner. It is expected that the functional assessment of the upper body, especially dynamic variables, can provide additional information for complementing diagnostic imaging and decision making during clinical daily routine.
